# ‘Shades of grey’: a focus group study on diagnostic uncertainty among general practitioners using point-of-care ultrasound

**DOI:** 10.1080/02813432.2024.2423242

**Published:** 2024-11-06

**Authors:** Hans-Christian Myklestul, Holgeir Skjeie, Mette Brekke, Trygve Skonnord

**Affiliations:** a Department of General Practice, Institute of Health and Society, University of Oslo, Oslo, Norway; b General Practice Research Unit (AFE), Department of General Practice, Institute of Health and Society, University of Oslo, Oslo, Norway

**Keywords:** Clinical decision-making, ultrasonography, doctor patient relation, family practice, interdisciplinary communication, uncertainty

## Abstract

**Background:**

Point-of-care ultrasound (POCUS) has long been a diagnostic tool in family medicine, although most Norwegian general practitioners (GPs) who use POCUS, scans infrequently. The broad scope of family medicine, the relatively low prevalence of illnesses and infrequent use of POCUS imply that GPs may experience diagnostic uncertainty regularly.

**Aim:**

To explore how GPs perceived and managed diagnostic uncertainty when using POCUS.

**Design and setting:**

A qualitative focus group study among Norwegian GPs using POCUS.

**Methods:**

Four focus group discussions were conducted. Total number of participants were 21. The interview guide was piloted, the focus group discussions were audio-recorded and transcribed, and Systematic Text Condensation, a thematic cross-case analysis, was used to analyse the data.

**Results:**

Diagnostic uncertainty using POCUS was considered as aligning to the general clinical uncertainties in family medicine, but there were also POCUS-specific uncertainties in clinical decision-making. We generated six themes: emotional, cognitive, and ethical uncertainty using POCUS, communicating uncertainty to patients, interaction with specialists when using POCUS, and coping strategies of participants. POCUS results were the only results the participants sometimes withheld when communicating with other specialists. POCUS itself stimulated a renewed interest in family medicine. Scanning enough patients was the recommended coping strategy.

**Conclusion:**

POCUS-using GPs experienced diagnostic uncertainty when using POCUS that aligned with other diagnostic uncertainties they experienced in everyday practice. However, they did not treat the results like other findings, as the GPs at times withheld their POCUS findings when interacting with secondary care specialists. This requires further investigation.

## Introduction

General practitioners (GPs) use point-of-care ultrasound (POCUS) for a wide variety of examinations [1–6]. Norwegian GPs have used POCUS since the early 1980s [7–9], and nearly 40% of Norwegian GPs scanned in 2016 [10]. Most GPs use POCUS as a focused examination to rule-in or rule-out a clinical condition based on patients’ history, and physical and laboratory findings [5,11]. GPs may scan a variety of conditions from simple to moderate complexity with a high level of precision [5,12–15]. However, some GPs perform exploratory scans on patients without a suspected clinical condition [5,16].

Currently, there is no curriculum for POCUS in the post-graduate training of Norwegian GPs. The American Academy for Family Physician has developed a suggested guideline for the development of curricula of American family medicine residency programs [17]. Consensus-based lists exist for which scans GPs can and may do [18–21]. These lists were made from either two-step or three-step Delphi processes, where experienced GPs who used POCUS were asked which scans were considered most useful. A summary of recommendations can be found in Supplementary File 1.

Diagnostic uncertainty is an integral part of clinical reasoning and decision-making. This holds especially true in primary care, where patients come in various stages of diseases and often before specific symptoms of the disease are present, as displayed by Malterud et al. [22] and Stolper et al. [23] in their theoretical articles on the complexity of decision-making in general practice. There is no definition of diagnostic uncertainty that is agreed upon, nor is there a framework for measuring this. In a systematic review of diagnostic uncertainty, Bhise et al. described diagnostic uncertainty as an emotional response to clinically complex situations and suggested the following definition: ‘a subjective perception of an inability to provide an accurate explanation of the patient’s health problem’ [24].

The scope of family medicine is broad, where symptoms are common, and the frequency of each specific disease is relatively low [25–27]. A recent study showed that three out of four scanning GPs in Norway scanned less than 10 times annually [10]. The infrequent use of POCUS in general practice implies that maintaining the required skills may be challenging. Given the presence of the patient, GPs must give the patient feedback on the ultrasound scan. In cases where there is non-alignment between the patient history and clinical findings, GPs must balance the potentially harmful consequences of delayed diagnosis with the problem of over-diagnosis and overtreatment as described by Alam et al. [28]. OECD described in 2017 that one in ten patients is unnecessarily harmed at the point of care [29], due to lack of access to the GP or variation in adherence to guidelines leading to unnecessary CT scans for low back pain or inappropriate antimicrobial prescriptions.

In this study, we explored how GPs react when patient history, physical or laboratory exams and POCUS results do not correspond. We used the study by Alam et al. on the management of diagnostic uncertainty in primary care as inspiration for the development of the interview guide [28]. ‘The Reflective Practitioner’ by Schön served as a substantive theory for our interpretation of the results [30]. The research question for this study was: ‘How do GPs experience and manage uncertainty when using POCUS?’.

## Material and methods

### Sampling participants

Participants were selected by purposive sampling, focusing on spread in geographical locations. The first approach was an invitation *via* two Facebook© fora, one open to all Norwegian GPs and the other for members of the national GP POCUS association. This strategy failed to recruit any participants. Subsequently, GPs were identified by cross-matching the GP POCUS association membership list with the list of GPs in Norway. Among these, GPs in surrounding municipalities to the planned discussions received individual invitations *via* Facebook Messenger©. The focus groups were assembled by HCM for these discussions. Invitations and responses are recorded in Table 1 and a description of the participants is given in Table 2. The participants were informed about the purpose of the study and the research experience and POCUS experience of the interviewers prior to the focus groups. The interviewers did not account for their biases and assumptions. All participants gave their written consent to participate prior to the discussions.

**Table 1.** Invited GPs to participate in focus group interviews.

					
Localisation	Group 1Large city >100.000 inhabitants	Group 2Rural area	Group 3Mid-sized town 50–100.000 inhabitants	Group 4Mid-sized town 50–100.000 inhabitants	Sum
Participated	6	5	5	5	21
Not able to participate	5	6	5	18	34
Maybe later	6	0	2	3	11
Did not answer	20	12	12	16	60
Other	1	1	0	0	2
Sum	38	24	24	42	128

**Table 2.** Characteristics of participating GPs 2022.

Category	Variable	n
Sex	Male	12
	Female	9
Age	Years	34 – 67
Experience in clinical practice	Years	3 – 40
Specialist in family medicine	Yes	17
	No	4
Experience with POCUS	Years	<1 – 10
Average frequency of scanning*	Several times daily	4
	Once or twice daily	5
	1-4 times weekly	9
	1-3 times monthly	2
	Rarer	0

* One of the participants did not answer this question.

### The interview guide

We used an interview guide (Box 1) adapted from Alam et al. [28], which was piloted by HCM in a group of 11 GPs that used POCUS. Alam et al. wrote a systematic critical review on how diagnostic uncertainty is managed in primary care. They described how GPs can experience an interplay of cognitive, emotional, and ethical reactions as a response to diagnostic uncertainty. Within the cognitive domain, factors such as knowledge and a supportive environment, the GPs’ epistemological stance (biomedical vs biopsychosocial model), and personality traits were described as factors affecting the management of diagnostic uncertainty. Within the emotional domain, acceptance of diagnostic uncertainty in family medicine, clinical decision-making including intuition and gut feeling, personality traits as well as the GPs’ epistemological stance were described as relevant factors. Within the ethical domain, patients’ reactions to ambiguity, and the GPs’ clinical experience and personality traits were described as relevant factors in the management of diagnostic uncertainty. We modified the interview guide according to the feedback from the pilot discussion to ensure that the questions were relevant to the research question. Data from this discussion were not included in the analysis.

### The focus group discussions

HCM made a schedule for each discussion; each lasted approximately 1 hour 15 min during which we took brief field notes. In total, four focus-group discussions were held in the period January to March 2022, each with five or six participants. HCM and HS guided the first discussion, while HCM and TS guided the last three discussions. The discussions were audio-recorded and transcribed verbatim by HCM after each discussion. The data was stored on a server at the University of Oslo. The facilitators evaluated the discussion after each focus group. The participants did not receive a transcript of the discussion. Repeat discussions were not conducted. The first three discussions were held at GP clinics, while the last was held at a meeting facility. After the fourth focus group, the research group had a dialogue concluding that we had reached sufficient information power, as outlined by K. Malterud [31]. HCM, HS, and TS are male, and MB female. HCM is a Ph.D. student. HS, TS, and MB are researchers with experience in qualitative research. All four are clinically experienced specialists in family medicine. HCM was the only user of POCUS in the research group, with experience since 2015.

### Data analysis and theory

**Figure 1.**
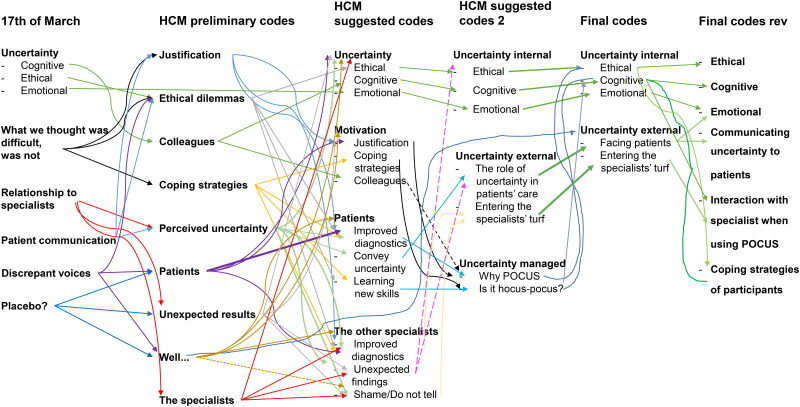
Flow chart of the coding process.

We used systematic text condensation to analyse the transcribed focus group interviews [32]. This method for thematic cross-case analysis of texts consists of four steps [1]: reading all the material for an overall impression and to elicit preliminary themes [2], making code groups from the preliminary themes while identifying meaning-bearing units describing the GPs’ experiences of uncertainty while using POCUS [3], proposing subgroups that exemplify central aspects of each code group, condensation of the contents of each subgroup, and finding quotes to be used for illustration, and [4] resynthesizing the condensates from each of the code groups so that the material can be presented on how the GPs experienced uncertainty using POCUS. HCM, TS, HS, and MB performed step [1]. For step [2], HCM and TS made the initial code groups for the first two interviews, and HCM made code groups for the last two interviews. In step [3] of the analysis, HCM proposed subgroups for each code group. The research group then edited the subgroups through discussions. HCM resynthesized the condensates in the last step [4] and drafted the initial report, which underwent internal review in the group before being finalized. The study by Alam et al. was used as a guide during the analysis [28]. The entire research group evaluated each step of the analytic process. NVivo qualitative data analysis software version 12 (QSR International Pty Ltd) was used to organize the material. A coding tree was not used for the analysis. However, a flow chart for the coding process is presented in Figure 1. One participant from each discussion received a copy of the results for feedback. No one raised any objections to the results. Consolidated criteria for reporting qualitative research (COREQ) was used as a checklist for this report [33].

In his book ‘The Reflective Practitioner’, the American social scientist Donald A. Schön approaches the epistemology of what experienced professionals actually do when they claim they know more than they can say, a tacit knowing-in-action [30]. In our understanding, this holds especially true for family medicine. When GPs merge their medical expertise and clinical experience with patients’ histories and symptoms, they need to reflect on their intuitive knowing amid action and use this capacity to cope with the unique, uncertain, and conflicted situation of everyday practice. Alam et al. described similarly, how GPs use a variety of cognitive, emotional, and ethical tools to manage diagnostic uncertainty [28]. The use of Schön as a substantive theory is supported by studies by Stolper et al. [23], Malterud et al. [22], as well as Cairo Notari et al. [34], to display the complex process of clinical decision-making, including patient-related, GP related as well as interactional dimensions, and adherent diagnostic uncertainty. We used Schön’s theory to support the analysis, and as a reflection in the discussion of the results.

The regional ethical committee assessed that the project did not need approval on 17 Nov 2020, application number 207905. The Norwegian centre for research data approved the project on 23 Nov 2020, approval number 323591.

## Results

In a reflexive and explorative analysis, we regenerated and found alignment in the categories described by Alam et al. In their review, they concluded that diagnostic uncertainty in family medicine could be conceptualized into emotional, cognitive, and ethical categories [28], and the participants in the present study confirmed and nuanced these concepts. In addition, in the reflexive analysis we developed POCUS-specific categories for perceived uncertainty, a notion that we were unaware of prior to the analysis. We categorized them as communicating uncertainty to patients and interacting with the specialists. Furthermore, in all four interviews, the participants suggested various coping strategies closely related to their experience of uncertainty, which we placed in a separate category called coping strategies of participants. Although more than one of these categories could be present at the same time, our interpretation emphasized the various aspects of each dimension.

The analysis developed a few findings that overarched the various categories. Many participants perceived POCUS as a powerful tool, that improving their diagnostic skills. As an additional finding, several participants reported spontaneously that POCUS had created a new interest in family medicine, and many enjoyed scanning, having a look into patients’ bodies. Some participants had experienced patients being positively surprised when the GP performed POCUS. We now elaborate on our assessments.

### Emotional dilemmas with the use of POCUS

Several participants expected that scanning would give clear-cut answers and reported a fear of doubt in their skills not being able to interpret their findings. Equivocal findings led to a rush of thoughts on the significance of them, and how the finding would affect patient follow-up, as well as how to convey this information to the patients. Some also reported a sense of shame if their scans did not fulfill the expectation of clear-cut answers. As one of the participants said:

*‘Occasionally when I scan a patient, I have no idea what is going on. There I sit, staring out and ask myself ‘Why did I do this? What am I doing?’ Because this means I must call the specialist. I have to say to the specialist that I do not know. Or if I can trust my own scans. These situations are so … uncomfortable.’ Harry, age 47*

There was a general conception among the participants that their initial scans were perceived as more uncertain compared with those scans made when they were more experienced users of POCUS. One participant emphasized the importance of maintaining a professional façade towards the patient.

### Cognitions about the benefits, harms, and doubts of POCUS

Quite a few of the participants frequently perceived results from scans as more uncertain compared with other examinations due to a lack of both formal and practical skills. One participant emphasized that POCUS was not part of the curriculum in medical school. The threshold was high regarding trust in their findings using POCUS due to the aforementioned lack of skills, especially when they were inexperienced scanners. Ruling-out a condition was considered by a few to be more difficult compared to ruling-in. Several of the participants also stated that patients differ, as some are easier to scan than others are. As one participant said:

*‘The machine does its job; we must cope with uncertainty. On the other hand, that is nothing new. Family medicine is often uncertain. Ultrasound is no different.’ Christopher, age 54*

Most participants relied on clinical findings rather than on POCUS findings when the patient’s story, physical examinations and scan results did not correspond. Most of the participants stated that there were uncertainties in all examinations performed in general practice, and their experiences using POCUS were no different.

*‘Basically, there is no difference, compared to the uncertainty linked to everything else we are doing. No, everyday practice is uncertain. When you auscultate the heart, there is an uncertainty to that procedure as well. You cannot know if you hear every sound. Is there a difference to that uncertainty when you scan? I do not know. Should you act upon that lab result? Or what does that pain mean? That stomach-ache, can it be something else? There is always some uncertainty. We must take all that into account in clinical assessment.’ Rosie, age 37*

### Ethical dilemmas using POCUS

Several of the participants emphasized the importance of scanning many patients to be able to master POCUS. This included scanning outside of the strict indications for doing specific scans. In turn, this may give occasional findings with unknown clinical significance, such as thyroid nodules when scanning the carotids for plaques and may lead to more examinations. Several of the participants accepted this as a necessary consequence of what otherwise might be seen as a dubious practice, although a few found this troublesome. One participant commented:

*‘I am thinking that it is not whether you should perform an ultrasound scan or not, because if you want to become good at it, you must use it a lot. But those ‘Choosing wisely’ concerns, one ought to be cautious to the ways one handles accidental findings.’ Ursula, age 37*

A few described ethical dilemmas about informing the patient after scanning pregnant women. One of the participants described his experience:

*‘I scanned a pregnant woman and found two dead foetuses, estimated early week 10. I had to make up my mind, should I inform her there and then, or refer her to the hospital? It was just after I had started using POCUS and I was in doubt. I mean, it would be brutal to tell her. Especially if she still was pregnant. I wanted the obstetrician to convey the information. So, I referred her. She was upset afterwards, that I had identified the two dead foetuses and not informed her. I just wanted that they told her in the hospital.’ Luke, age 39*

### Communicating the ‘shades of grey’ to patients

Several participants reported the dilemma of informing patients about what they found in their scans when they lacked confidence. There was a concern that giving patients incorrect information, such as suspected cancer or a slightly dilated aorta could harm the patient unnecessarily. On the other hand, many participants reported that they had experience in communicating uncertainty to patients, such as high blood pressure or elevated blood glucose levels, and that uncertainty after the use of POCUS was no different. One said, concerning a potential serious finding:

*‘There are shades of grey. There are some [patients] that are impossible to scan well. Then you must … be able to communicate uncertainty without scaring the living daylights out of the patient.’ Eric, age 41*

### Skirmishes on the specialists’ turf

Many participants had experienced negative feedback from colleagues in hospitals after using POCUS, and several described a perceived lack of trust from hospital specialists in the GP’s sonographic assessment. The notion of ‘turf battle’ came up, and some participants reported episodes of being reproached for not knowing the correct radiological terms when describing suspected pathology. As one participant said,

*‘I do think there is something striking about those emotions. Especially when you are communicating with other [specialists] about your findings. There is a sense of shame, because you have done an examination for which you did not have sufficient indication. Maybe you were not good enough?’ Rita, age 44*

Some participants were told by patients that the specialist said to the patient ‘*anyone could see that*…’.

Several participants reported the experience that specialists perceived their scans as worthless, and that their findings were not worth storing in the patient’s electronic medical records; thus, several participants said they would remove POCUS results from referrals. These results were the only ones, of any examination made, they withheld. In the words of two of the participants:

*‘I have referred patients without describing results from scanning. I do not dare to write. I find that odd. However, I have met condescension [from hospital doctors]. Thus, I avoid reporting sonographic results.’ Xenia, age 54*

‘*I feel this unexpressed attitude [towards GPs who scan] from the specialists at the hospital. They look down on us, in family medicine. But they do not know our practices, and what we do.’ Xandra, age 47*

In contrast, some reported an increased acceptance from hospital doctors when GPs used POCUS.

### Mastering POCUS: coping strategies of participants

Many participants assessed POCUS to improve diagnostics, with scanning for a foetal position at a term considered better than Leopold’s manoeuvres as a frequently used example. Scanning numerous patients was a coping strategy for mastering a new skill that was adopted universally. Enough time to scan and the necessity of investing time to learn were other coping strategies adopted by some of the participants. The participants considered ruling-in easier than ruling-out. The participants recommended a humble approach when entering a new field, due to the complexity of family medicine. This also applied to POCUS.

Another coping strategy was to reduce patients’ expectations prior to scanning. Almost all participants used this, in reference to both their skills and the equipment available, with statements such as *‘I am a GP, not a radiologist’ and ‘The machine I have is inferior to that of the hospital’.* The participants found that the patients accepted such comments, and this attitude from the patients reduced stress for the participants whenever they could not find the organs they were scanning. In the words of one of the participants:

*‘I usually think I see what I see, and that is okay. What I do not see, however… It may be there; I just cannot find it. Then uncertainty hits me. Then again, I think as if I did not have an ultrasound, all right? And then, I acted as if I did not have ultrasound. I rely on the clinical findings and all the other tools I have as a part of clinical assessment.’ Xavier, age 48*

## Discussion

### Summary

All participants experienced uncertainty when using POCUS, but this experience was not perceived as qualitatively different compared with the uncertainty experienced when analysing a patient’s history and physical and laboratory examinations. However, there was a discrepancy between what the participants said and what they did. POCUS results were the only patient-related information they withheld when interacting with specialists, including referrals. Six categories related to uncertainty were identified: emotional, cognitive, ethical, communicative, and interactional dimensions, and coping strategies of participants. The participants enjoyed scanning patients, and the use of POCUS caused a renewed interest in family medicine.

### Strengths and limitations

The participants and researchers in this study were all GPs. Two GPs, one with and one without POCUS knowledge facilitated all discussions. As all participants were GPs, including the researchers, this provided a safe setting for the discussions. However, bringing in a non-physician to the discussion might have led to questions and answers on issues taken for granted by GPs, thereby reducing possible confirmation and response biases. We used an interview guide based on a systematic review on diagnostic uncertainty in primary care by Alam et al. [28]. The interview guide was piloted on a group of GPs who used POCUS and modified to make sure the questions could be used to answer the research question.

Focus groups were used to gather data for this study. This method is considered feasible to organize [35, 36], and the discussion between the participants of the group can bring forth experiences and opinions from participants who would not engage in individual interviews [35]. When reviewing the discussions in hindsight, we saw that we ought to have challenged the term ‘shame’. Rather, a sense of ‘guilt’ might have been more appropriate; the less pervasive effect of this emotion may have been more precise. On the other hand, as the participants were native Norwegian speakers, we assumed that they mastered the language.

We used purposive sampling to invite GPs who used POCUS [37]. Even though the groups were assembled for this study, participants in each group knew about each other and felt comfortable discussing the issues of uncertainty, and they commented on each other’s experiences of uncertainty. Physical meetings further facilitated the free flow of conversation, and the interviewers attempted to split talk time relatively equally among the participants. Given that our participants practiced scanning more frequently than most GPs in Norway [10], this weakens the external validity. However, they were heterogeneous in terms of age, gender, years in practice and POCUS experience as well as practice location (rural/urban), all of which strengthened the transferability of our results.

We chose a descriptive and interpreting phenomenological-inspired thematic cross-case analysis for this study because the design is well suited for exploring clinical experiences [32,38,39].

The information power of the study was considered sufficient, based on four of the five terms set by Malterud et al. [1, 31]: experienced uncertainty when using POCUS was considered a narrow aim [2]; the sample specificity was dense, as all participants except one were frequent users of POCUS [3]; a substantive theory was used for the interpretation of data [4]; the dialogue was characterized by active participants and clear communication [5]; this study was a cross-case analysis, which requires more participants. With four focus groups of five and six participants in each, we considered the number of participants to be sufficient.

### Comparison with existing literature

The introduction of POCUS in family medicine has been a technologically driven evolution of practice, as a clinical tool for both diagnostic and therapeutic purposes. Medicine was refashioned in the light of positivism during the twentieth century [30], as Schön notes in ‘The Reflective Practitioner’. As evidence-based medicine evolved and was applied in clinical practice, the dilemma of rigor versus relevance arose. In some fields of medicine, Schön notes that clinicians could take a position on the high ground effectively using research-based theory and techniques, whereas others chose the ‘swampy lowlands where situations are confusing ‘messes’ incapable of technical solutions’ [30](p42). This holds for family medicine. White described the common presence of symptoms of disease in the adult population in the article ‘The ecology of health care’ [25]. These results have later been replicated [26,27]. GPs must decide which patients have a disease, and which patients are those with illness, but without a specific disease. McWhinney described the differences between hospital and general practice regarding decision-making [40]. Patients experience transient symptoms or see their GP in the initial stages of disease, when the disease has not evolved, or the common symptoms have not appeared. Stolper et al. [23], Malterud et al. [22], and Cairo Notari et al. [34] described in their studies the GPs’ complex clinical reasoning processes balancing risks and benefits of adhering to guidelines while they are simultaneously prioritising what is considered the best for their patients.

The participants assessed POCUS as a powerful diagnostic tool. We aimed to examine experienced uncertainty when GPs use POCUS, and not to assess whether POCUS was considered a useful diagnostic tool. However, participants spontaneously reported the experience of improved quality of their clinical assessments, for example, by comparing POCUS to Leopold’s manoeuvres when assessing foetal position at term. Similar results have been reported when interviewing GPs about their use of POCUS [4,16,41].

In addition, the participants experienced the use of POCUS as fun and noted that it had renewed their interest in family medicine. Andersen et al. found that GPs who used POCUS experienced ‘increased job enthusiasm and professional contentment’ [16,42]. Schön described how reflective practitioners who move towards new skills give up the notion of unquestioned authority and gain new satisfaction in their knowledge-in-practice and in themselves [30]. In turn, the practice itself becomes a source of renewal. ‘Recognition of error, with its resulting uncertainty, can become a source of discovery rather than an occasion for self-defense’ Schön further states. Cunningham and Wilson argue that in practices where error becomes a source of discovery rather than self-defense, shame is replaced with curiosity, and experienced as beneficial by the GP [43].

#### The emotional dimension

In the emotional dimension of diagnostic uncertainty, several of the participants described their expectations of clear-cut answers when scanning patients and the emotional stress of equivocal findings. Alam et al. described how the GPs’ gender, personality traits, clinical experience and epistemological stance affect the emotional management of uncertainty [28]. We did not find gender-specific variations in the management of emotional uncertainty when the GPs used POCUS, nor did we probe the question of personality traits. However, the participants confirmed the positive effects of experience with POCUS on the emotional dilemmas scanning patients. Evans and Trotter found in their article that a biomedical epistemology was associated with higher, and a biopsychosocial epistemology was associated with a lower stress reaction to diagnostic uncertainty [44]. The former epistemological stance may explain the expectations and following emotional stress of ambiguous scans. A few of the participants even described a sense of shame when scans could not answer their clinical questions, possibly because of the positivistic paradigm of technical rationality in medicine, and not being able to fulfil the role of an expert [30](p300). Cunningham and Wilson described how shame may negatively affect the clinician’s behaviour when having to face their own lack of knowledge [43].

#### The cognitive dimension

In the cognitive dimension, the participants reported more uncertainty when they scanned patients compared to other examinations due to lack of skills and experience. Geis et al. described a similar lack of confidence in their skills when Swiss GPs were interviewed about their use of POCUS as a part of pneumonia diagnosis [45]. Alam et al. described how GPs benefited from clinical experience and minimizing knowledge gaps to manage the cognitive dimension of diagnostic uncertainty [28]. Another coping strategy described by Alam et al. was safety netting, in which our participants reported relying on clinical findings rather than POCUS when the results did not correspond. Cox et al. also described the same strategy among GPs as a cognitive strategy to manage diagnostic uncertainty [46]. Ruling-out conditions were reported by our participants as more difficult. Aakjær Andersen et al. found that most Danish GPs used POCUS to rule-in or rule-out clinical conditions [5], but when Andersen et al. interviewed a group of Danish GPs, a few argued that GPs should not use POCUS to rule-out clinical conditions [47].

#### The ethical dimension

In the ethical dimension, the participants experienced dilemmas when informing patients about their uncertainty using POCUS. Conveying emotionally laden information to patients based on their scans, such as lack of heart activity in a foetus, was deemed challenging by the participants, especially when it could imply a major crisis for the patient. Alam et al. found that inexperienced GPs were reluctant to disclose uncertainty to patients, which was part of the case here [28]. Cox et al. described the ethical dimension of diagnostic uncertainty in primary care as a subcategory to communicating uncertainty to patients [46]. They described the harmful effect of not discussing diagnostic uncertainty with patients, that our participant also had experienced.

#### The dimension of communication

Communicating uncertainty to patients was reported as challenging, as it could harm patients. Alam et al. found that GPs assessed their patients’ responses to uncertainty before they shared their ambiguity [28]. Cox et al. described a similar intolerance to uncertainty among patients as one factor limiting the communication of uncertainty [46]. They further addressed how communicating diagnostic uncertainty to patients can both improve and reduce trust in the patient-doctor relationship and identified this as a vital skill in primary care. Although the reflective contract demands practitioners with high levels of competency, the reflective contract in the patient-doctor relationship gives more responsibility to the patient as an active participant according to Schön [30](p302). The reflective contract we interpreted as the long-term relationship between the patient and the GP. Cox et al. further describe how insufficient communication of diagnostic uncertainty disempowers patients in shared decision-making [46]. On the other hand, many of our participants reported experience in communicating uncertainty to patients.

#### The dimension of coping

Coping with diagnostic uncertainty using POCUS in primary care was reported in many ways. The participants attempted to reduce patients’ expectations of their skills and available equipment prior to the scans, which contradicted to the perceived expert role of physicians [30](p300). POCUS was the only examination in which the participants devaluated their skills and equipment to the patients. This surprising and interesting finding suggests further investigations for potential causes for this behaviour. Andersen et al. described a similar behaviour among Danish GPs using POCUS [16]. In a study by Akanuwe et al. British GPs further stated an attitude that ‘community POCUS should complement the work of radiologists rather than replace them’ [48]. For a reflective practitioner not needing to maintain a professional façade, such an attitude may improve the patient-doctor relationship. The positive feedback some of the participants had received, was previously shown by Andersen et al. [49]. They reported that patients assessed examinations as more thorough when their GP used POCUS and that the use of POCUS improved the doctor-patient relationship for almost half of the patients.

Scanning a sufficient number of patients was another strategy adopted by most of the participants to master POCUS. Similar strategies have been reported by GPs elsewhere [41,45,48]. However, little time is necessary to invest to master several focused scans [14,15,50–55]. On the other hand, our participants’ desire to learn made them scan outside the available recommendations. Andersen et al. found that more than 10% of the reported scans were made outside the curriculum taught to Danish GPs [3]. A study by Aakjær Andersen et al. found that more than 20% of scans by GPs were exploratory without a suspected clinical condition [5]. However, this practice was considered dubious by some participants and created ethical challenges about ‘choosing wisely’ when they encountered incidental findings that might lead to a cascade of events that were potentially harmful to patients as well as imposing a financial burden on them, as previously described by Ganguli et al. [56]. Akanuwe et al. reported similar worries among British GPs for incidental discoveries and unintended consequences when using POCUS [48]. Cox et al. also described an ethical dilemma with respect to patients’ autonomy in informed shared decision-making on the one hand, and the responsibility of GPs on health resource management on the societal level on the other [46].

#### The interactive dimension with secondary care specialists

Many participants had experienced negative feedback from secondary care specialists. Mengel-Jorgensen and Bach Jensen described scepticism in the medical community as one of many barriers to the implementation of POCUS in primary care [1]. Müller et al. have described how disparaging comments from a specialist care doctor negatively affected a GP [57]. As a result, participants may have withheld information about POCUS results, but not other clinical information. This finding was a surprising and interesting result. To our knowledge, the attitudes of secondary care specialists towards GPs using POCUS have not been addressed in research. Nor have we found research on GPs deliberately withholding clinical information when referring patients to specialists. The participants said they experienced uncertainty when using POCUS as no different compared to any other uncertainty in family medicine. However, they certainly managed it differently. Schön commented that some professionals opt for the high ground of medicine, ‘devoted to an image of solid professional competence’ [30], whereas GPs practice in the lowlands of clinical medicine. This may explain our results.

### Implication for research and practice

According to our participants, uncertainty using POCUS was not experienced as different from other uncertainties in family medicine, though there were POCUS-specific angles in the uncertainties. One area that requires investigation is why the participants sometimes deliberately withheld POCUS results when interacting with specialists. This was the only patient information the participants withheld. This behaviour may be related to the specialists’ potentially negative feedback and a potential following sensation of shame second to that experience. The experience of improved diagnostics may have counterbalanced this negative emotion; nevertheless, this issue requires further examination. Secondary care specialists’ attitudes towards GPs who use POCUS is another topic that needs to be elucidated. In the future, research is needed on the appropriate use of POCUS in family medicine, and the training that is required to learn and master scans of various organs to be professionally sound in a GP setting, on how GPs can best learn and use the ‘shades of grey’.

## Supplementary Material

Supplemental Material

24 08 13 Supplementary file 1.docx

## References

[1] Mengel-Jørgensen T, Jensen MB. Variation in the use of point-of-care ultrasound in general practice in various European countries. Results of a survey among experts. Eur J Gen Pract. 2016;22(4):274–277. doi: 10.1080/13814788.2016.1211105. 27487159

[2] Andersen CA, Holden S, Vela J, et al. Point-of-care ultrasound in general practice: a systematic review. Ann Fam Med. 2019;17(1):61–69. doi: 10.1370/afm.2330. 30670398 PMC6342599

[3] Andersen CA, Frandsen AK, Valentiner-Branth C, et al. Introducing point-of-care ultrasound in Danish general practice-elucidating the use through a medical audit. Fam Pract. 2021;38(2):80–87. doi: 10.1093/fampra/cmaa080. 32839822

[4] Touhami D, Merlo C, Hohmann J, et al. The use of ­ultrasound in primary care: longitudinal billing and cross-sectional survey study in Switzerland. BMC Fam Pract. 2020;21(1):127. doi: 10.1186/s12875-020-01209-7. 32611390 PMC7330951

[5] Aakjær Andersen C, Brodersen J, Davidsen AS, et al. Use and impact of point-of-care ultrasonography in general practice: a prospective observational study. BMJ Open. 2020;10(9):e037664. doi: 10.1136/bmjopen-2020-037664. PMC7500300 32948563

[6] Nathanson R, Williams JP, Gupta N, et al. Current use and barriers to point-of-care ultrasound in primary care: a national survey of VA medical centers. Am J Med. 2023;136(6):592–595.e2. doi: 10.1016/j.amjmed.2023.01.038. 36828205

[7] Bratland SZ. [Ultrasonic diagnosis in general practice. An evaluation study]. Tidsskr Nor Laegeforen. 1985;105(28):1939–1940. 3907001

[8] Eggebø TM, Dalaker K. Ultralydundersøkelser av gravide i allmennpraksis [Ultrasonic diagnosis of pregnant women performed in general practice]. Tidsskr Nor Laegeforen. 1989;109(29):2979–2981. 2686093

[9] Johansen I, Grimsmo A, Nakling J. [Ultrasonography in primary health care–experiences within obstetrics 1983-99]. Tidsskr Nor Laegeforen. 2002;122(20):1995–1998. 12555445

[10] Myklestul HC, Skonnord T, Brekke M. Point-of-care ultrasound (POCUS) in Norwegian general practice. Scand J Prim Health Care. 2020;38(2):219–225. 32314640 10.1080/02813432.2020.1753385 PMC8570758

[11] Phillips H, Sukheja N, Williams S, et al. Point-of-care ultrasound in general practice: an exploratory study in rural South Australia. Rural Remote Health. 2023;23(1):7627. doi: 10.22605/RRH7627. 36792605

[12] Lindgaard K, Riisgaard L. Validation of ultrasound examinations performed by general practitioners. Scand J Prim Health Care. 2017;35(3):256–261. doi: 10.1080/02813432.2017.1358437. 28776457 PMC5592352

[13] Diprose W, Verster F, Schauer C. Re-examining physical findings with point-of-care ultrasound: a narrative review. N Z Med J. 2017;130(1449):46–51. 28178729

[14] Mai T, Woo MY, Boles K, et al. Point-of-care ultrasound performed by a medical student compared to physical examination by vascular surgeons in the detection of abdominal aortic aneurysms. Ann Vasc Surg. 2018;52:15–21. doi: 10.1016/j.avsg.2018.03.015. 29777851

[15] Decara JM, Kirkpatrick JN, Spencer KT, et al. Use of hand-carried ultrasound devices to augment the accuracy of medical student bedside cardiac diagnoses. J Am Soc Echocardiogr. 2005;18(3):257–263. doi: 10.1016/j.echo.2004.11.015. 15746716

[16] Andersen CA, Davidsen AS, Brodersen J, et al. Danish general practitioners have found their own way of using point-of-care ultrasonography in primary care: a qualitative study. BMC Fam Pract. 2019;20(1):89. doi: 10.1186/s12875-019-0984-x. 31253102 PMC6599254

[17] Physicians AAoF. Recommended Curriculum Guidelines for Family Medicine Residents Point-of-Care Ultrasound 2016 [Available from: https://www.aafp.org/dam/AAFP/documents/medical_education_residency/program_directors/Reprint290D_POCUS.pdf.

[18] Løkkegaard T, Todsen T, Nayahangan LJ, et al. Point-of-care ultrasound for general practitioners: a systematic needs assessment. Scand J Prim Health Care. 2020;38(1):3–11. doi: 10.1080/02813432.2020.1711572. 31955658 PMC7054965

[19] Camard L, Liard R, Duverne S, et al. Consensus on relevant point-of-care ultrasound skills in general practice: a two-round French Delphi study. BMC Med Educ. 2024;24(1):341. doi: 10.1186/s12909-024-05072-3. 38532436 PMC10967120

[20] Homar V, Gale ZK, Lainscak M, et al. Knowledge and skills required to perform point-of-care ultrasonography in family practice - a modified Delphi study among family physicians in Slovenia. BMC Fam Pract. 2020;21(1):56. doi: 10.1186/s12875-020-01130-z. 32216753 PMC7098073

[21] Conangla-Ferrin L, Guirado-Vila P, Solanes-Cabús M, et al. Ultrasound in primary care: consensus recommendations on its applications and training. Results of a 3-round Delphi study. Eur J Gen Pract. 2022;28(1):253–259. doi: 10.1080/13814788.2022.2150163. 36503353 PMC9754009

[22] Malterud K, Reventlow S, Guassora AD. Diagnostic knowing in general practice: interpretative action and reflexivity. Scand J Prim Health Care. 2019;37(4):393–401. doi: 10.1080/02813432.2019.1663592. 31507239 PMC6883426

[23] Stolper E, Van Royen P, Jack E, et al. Embracing complexity with systems thinking in general practitioners’ clinical reasoning helps handling uncertainty. J Eval Clin Pract. 2021;27(5):1175–1181. doi: 10.1111/jep.13549. 33592677 PMC8518614

[24] Bhise V, Rajan SS, Sittig DF, et al. Defining and measuring diagnostic uncertainty in medicine: a systematic review. J Gen Intern Med. 2018;33(1):103–115. doi: 10.1007/s11606-017-4164-1. 28936618 PMC5756158

[25] White KL, Williams TF, Greenberg BG. The ecology of medical care. N Engl J Med. 1961;265(18):885–892. doi: 10.1056/NEJM196111022651805. 14006536

[26] Green LA, Fryer GE, Jr., Yawn BP, et al. The ecology of medical care revisited. N Engl J Med. 2001;344(26):2021–2025. doi: 10.1056/NEJM200106283442611. 11430334

[27] Hansen AH, Halvorsen PA, Forde OH. The ecology of medical care in norway: wide use of general practitioners may not necessarily keep patients out of hospitals. J Public Health Res. 2012;1(2):177–183. doi: 10.4081/jphr.2012.e28. 25180941 PMC4140360

[28] Alam R, Cheraghi-Sohi S, Panagioti M, et al. Managing diagnostic uncertainty in primary care: a systematic critical review. BMC Fam Pract. 2017;18(1):79. doi: 10.1186/s12875-017-0650-0. 28784088 PMC5545872

[29] OECD. Tackling Wasteful Spending on Health. Paris: OECD Publishing; 2017. doi: 10.1787/9789264266414-en.

[30] Schön DA. The Reflective Practitioner: how Professionals Think in Action (1st ed.). Routledge; 1992. doi: 10.4324/9781315237473.

[31] Malterud K, Siersma VD, Guassora AD. Sample size in qualitative interview studies: guided by information power. Qual Health Res. 2016;26(13):1753–1760. doi: 10.1177/1049732315617444. 26613970

[32] Malterud K. Systematic text condensation: a strategy for qualitative analysis. Scand J Public Health. 2012;40(8):795–805. doi: 10.1177/1403494812465030. 23221918

[33] Tong A, Sainsbury P, Craig J. Consolidated criteria for reporting qualitative research (COREQ): a 32-item checklist for interviews and focus groups. Int J Qual Health Care. 2007;19(6):349–357. doi: 10.1093/intqhc/mzm042. 17872937

[34] Cairo Notari S, Sader J, Caire Fon N, et al. Understanding GPs’ clinical reasoning processes involved in managing patients suffering from multimorbidity: a systematic review of qualitative and quantitative research. Int J Clin Pract. 2021;75(9):e14187. doi: 10.1111/ijcp.14187. 33783098 PMC8459259

[35] Kitzinger J. Qualitative research. Introducing focus groups. BMJ. 1995;311(7000):299–302. doi: 10.1136/bmj.311.7000.299. 7633241 PMC2550365

[36] Acocella I. The focus groups in social research: advantages and disadvantages. Qual Quant. 2012;46(4):1125–1136. doi: 10.1007/s11135-011-9600-4.

[37] Marshall MN. Sampling for qualitative research. Fam Pract. 1996;13(6):522–525. doi: 10.1093/fampra/13.6.522. 9023528

[38] Malterud K. The art and science of clinical knowledge: evidence beyond measures and numbers. Lancet. 2001;358(9279):397–400. doi: 10.1016/S0140-6736(01)05548-9. 11502338

[39] Järvinen M, Mik-Meyer N. Qualitative analysis: eight Approaches for the Social. Sciences SAGE; 2020.

[40] McWhinney IR. Problem-solving and decision-making in primary medical practice. Proc R Soc Med. 1972;65(11):934–938. 4642013 10.1177/003591577206501104 PMC1644732

[41] Sheppard G, Devasahayam AJ, Campbell C, et al. The prevalence and patterns of use of point-of-care ultrasound in Newfoundland and Labrador. Can J Rural Med. 2021;26(4):160–168. doi: 10.4103/cjrm.cjrm_61_20. 34643555

[42] Andersen CA, Brodersen JB, Graumann O, et al. Factors affecting point-of-care ultrasound implementation in general practice: a survey in Danish primary care clinics. BMJ Open. 2023;13(10):e077702. doi: 10.1136/bmjopen-2023-077702. PMC10582891 37848298

[43] Cunningham W, Wilson H. Shame, guilt and the medical practitioner. N Z Med J. 2003;116(1183):U629. 14581943

[44] Evans L, Trotter DR. Epistemology and uncertainty in primary care: an exploratory study. Fam Med. 2009;41(5):319–326. 19418279

[45] Geis D, Canova N, Lhopitallier L, et al. Exploration of the acceptance of the use of procalcitonin point-of-care testing and lung ultrasonography by general practitioners to decide on antibiotic prescriptions for lower respiratory infections: a qualitative study. BMJ Open. 2023;13(5):e063922. doi: 10.1136/bmjopen-2022-063922. PMC10186440 37169498

[46] Cox CL, Miller BM, Kuhn I, et al. Diagnostic uncertainty in primary care: what is known about its communication, and what are the associated ethical issues? Fam Pract. 2021;38(5):654–668. doi: 10.1093/fampra/cmab023. 33907806 PMC8463813

[47] Andersen CA, Guetterman TC, Fetters MD, et al. General practitioners’ perspectives on appropriate use of ultrasonography in primary care in Denmark: a multistage mixed methods study. Ann Fam Med. 2022;20(3):211–219. doi: 10.1370/afm.2795. 35606122 PMC9199035

[48] Akanuwe JNA, Siriwardena AN, Bidaut L, et al. Practitioners’ views on community implementation of point-of-care ultrasound (POCUS) in the UK: a qualitative interview study. BMC Health Serv Res. 2023;23(1):84. doi: 10.1186/s12913-023-09069-4. 36698100 PMC9876652

[49] Andersen CA, Brodersen J, Rudbæk TR, et al. Patients’ experiences of the use of point-of-care ultrasound in general practice - a cross-sectional study. BMC Fam Pract. 2021;22(1):116. doi: 10.1186/s12875-021-01459-z. 34144701 PMC8214303

[50] Steinmetz P, Oleskevich S, Dyachenko A, et al. Accuracy of medical students in detecting pleural effusion using lung ultrasound as an adjunct to the physical examination. J Ultrasound Med. 2018;37(11):2545–2552. doi: 10.1002/jum.14612. 29574857

[51] Kobal SL, Trento L, Baharami S, et al. Comparison of effectiveness of hand-carried ultrasound to bedside cardiovascular physical examination. Am J Cardiol. 2005;96(7):1002–1006. doi: 10.1016/j.amjcard.2005.05.060. 16188532

[52] Shmueli H, Burstein Y, Sagy I, et al. Briefly trained medical students can effectively identify rheumatic mitral valve injury using a hand-carried ultrasound. Echocardiography. 2013;30(6):621–626. doi: 10.1111/echo.12122. 23347259

[53] Panoulas VF, Daigeler AL, Malaweera AS, et al. Pocket-size hand-held cardiac ultrasound as an adjunct to clinical examination in the hands of medical students and junior doctors. Eur Heart J Cardiovasc Imaging. 2013;14(4):323–330. doi: 10.1093/ehjci/jes140. 22833550

[54] Mouratev G, Howe D, Hoppmann R, et al. Teaching medical students ultrasound to measure liver size: comparison with experienced clinicians using physical examination alone. Teach Learn Med. 2013;25(1):84–88. doi: 10.1080/10401334.2012.741535. 23330900

[55] Stokke TM, Ruddox V, Sarvari SI, et al. Brief group training of medical students in focused cardiac ultrasound may improve diagnostic accuracy of physical examination. J Am Soc Echocardiogr. 2014;27(11):1238–1246. doi: 10.1016/j.echo.2014.08.001. 25216765

[56] Ganguli I, Simpkin AL, Lupo C, et al. Cascades of care after incidental findings in a US National Survey of physicians. JAMA Netw Open. 2019;2(10):e1913325. doi: 10.1001/jamanetworkopen.2019.13325. 31617925 PMC6806665

[57] Müller BS, Donner-Banzhoff N, Beyer M, et al. Regret among primary care physicians: a survey of diagnostic decisions. BMC Fam Pract. 2020;21(1):53. doi: 10.1186/s12875-020-01125-w. 32183738 PMC7079478

