# Supplementary material for: ‘Shades of grey’: a focus group study on diagnostic uncertainty among general practitioners using point-of-care ultrasound
Source: Scand J Prim Health Care. 2024 Nov 6;43(1):219–29. doi: 10.1080/02813432.2024.2423242 (PMC11834800; doi:10.1080/02813432.2024.2423242)
Supplement: Supplemental Material [file IPRI_A_2423242_SM8992.docx]

**Supplementary data**

Box 1

**Interview guide**

**Main questions**

- How do you react if you cannot find what you are searching after?
- How do you handle uncertain findings?
- What do you say to the patient in case of uncertain findings?
- How do you deal with unexpected discoveries?

**In-depth questions**

- Which thoughts come to your mind in these situations?
- Which feelings can you recognize?
- Which ethical reflections do you do in these situations?

**Kick-off questions** (In case of hesitancy)

- Can you describe a situation where you could not find what you were searching after?
- Has any one of you had a similar experience?

We did not have to use the kick-off questions to get the discussions started.
