# Supplementary material for: ‘Shades of grey’: a focus group study on diagnostic uncertainty among general practitioners using point-of-care ultrasound
Source: Scand J Prim Health Care. 2024 Nov 6;43(1):219–29. doi: 10.1080/02813432.2024.2423242 (PMC11834800; doi:10.1080/02813432.2024.2423242)
Supplement: 24 08 13 Supplementary file 1.docx [file IPRI_A_2423242_SM3419.docx]

**Supplementary file 1**

Summary of recommended POCUS scans in primary care

| **Organ system** | **Lokkegaard et al. (1)** | | **Camard et al. (2)** | | **Homar et al. (3)** | | **Conangla-Ferrin et al. (4)** | |
| --- | --- | --- | --- | --- | --- | --- | --- | --- |
| **Lungs** | Pleural effusion | |  | | Pleural effusion | | Pleural effusion | |
|  |  | |  | | Pneumonia | | Pneumonic pulmonary condensation | |
|  |  | |  | | Pneumothorax | | Pneumothorax | |
|  |  | |  | | Pulmonary embolism | |  | |
|  |  | |  | |  | | Interstitial involvement of different causes (Interstitial pneumonia, diffuse interstitial lung disease), especially heart failure | |
|  |  | |  | |  | |  | |
| **Abdominal** | Gall stones | | Biliary lithias(es) | | Gallbladder disease, gallstones | | Cholelithiasis and cholecystitis | |
|  | Cholecystitis | | Cholecystitis | |  | |  | |
|  |  | | Dilation of the common bile duct | |  | |  | |
|  |  | | Hepatic dysmorphia | | Organomegaly | | Hepatomegaly and/or splenomegaly | |
|  |  | | Focal liver lesion | |  | |  | |
|  |  | |  | |  | | Ultrasound confirmation of abdominal mass to study and associated complications (adenopathy, abdominal free fluid, liver lesions…) | |
|  | Free abdominal fluid | | Peritoneal effusion | | Intra-abdominal free fluid | |  | |
|  |  | | Appendicitis | |  | | Appendicitis | |
|  |  | | Mesenterial adenitis | |  | |  | |
|  |  | | Constipation in children | |  | |  | |
|  |  | |  | |  | | Pancreatitis | |
|  |  | |  | | Ileus | |  | |
|  |  | |  | |  | | Abdominal wall hernia | |
| **Organ system** | | **Lokkegaard et al.** | | **Camard et al.** | | **Homar et al.** | | **Conangla-Ferrin et al.** |
| **Cardiovascular** | | Abdominal aortic aneurism | | Abdominal aortic aneurysm | | Abdominal aortic aneurism or dissection (screening) | | Abdominal aortic aneurysm |
|  | |  | |  | |  | | Aortic dissection |
|  | |  | | Diameter of abdominal aorta | |  | |  |
|  | |  | | Proximal iliac artery aneurysm | |  | |  |
|  | |  | |  | |  | | Determination of carotid intima media thickness |
|  | |  | |  | |  | | Presence of atheroma plaques and impact on carotid flow |
|  | |  | |  | |  | | Chronic venous insufficiency |
|  | | Deep venous thrombosis | |  | | Deep venous thrombosis | | Deep vein thrombosis |
|  | | Pericardial effusion | |  | | Pericardial effusion and heart tamponade | |  |
|  | |  | |  | | Heart size and motility | |  |
|  | |  | |  | |  | | Structural heart disease (combine with high cardiovascular risk study) |
|  | |  | |  | |  | | Decompensation of heart failure |
|  | |  | |  | | Evaluation of hydration (vena cava compliance) | |  |

| **Organ system** | **Lokkegaard et al.** | **Camard et al.** | **Homar et al.** | **Conangla-Ferrin et al.** |
| --- | --- | --- | --- | --- |
| **Gynaecological/Obstetrical** | Living intrauterine pregnancy | Intrauterine pregnancy | Pregnancy (confirmation, evaluation) | Gestation (verification of intrauterine gestation, verification of foetal position before imminent delivery), rule out associated complications (ectopic, multiple, retroplacental haematoma, absence of foetal heartbeat in advanced gestation…) |
|  | Gestational age (CRL measurement) | Gestational age of intrauterine pregnancy |  |  |
|  | Foetal position |  |  |  |
|  | First trimester bleeding |  |  |  |
|  |  | Foetal weight in second or third trimester of pregnancy |  |  |
|  |  | Amount of amniotic fluid in second and third trimester of pregnancy |  |  |
|  |  | Ectopic pregnancy | Extra-uterine pregnancy |  |
|  | Localization of intrauterine device | Location of intrauterine device |  |  |
|  |  | Intrauterine fibroids | Uterine myoma |  |
|  |  |  | Ovarian cysts |  |
|  |  | Adnexal torsion |  |  |

| **Organ system** | **Lokkegaard et al.** | **Camard et al.** | **Homar et al.** | **Conangla-Ferrin et al.** |
| --- | --- | --- | --- | --- |
| **Urogenital** | Bladder volume | Urine bladder volume |  |  |
|  |  | Post-micturition residue | Retention of urine | Urinary retention |
|  |  |  |  | Increased prostate volume and post-voiding residue |
|  |  |  | Cystitis |  |
|  |  | Bladder wall thickness |  |  |
|  |  | Bladder mass |  | Bladder tumour |
|  |  | Bladder diverticula |  |  |
|  |  | Bladder globe |  |  |
|  |  |  |  | Urinary tract malformation in patients with repeated urinary tract infections |
|  |  | Urinary lithiasis |  |  |
|  |  |  |  | Cystic injury with no warning signs |
|  | Hydronephrosis | Pyelocalyceal cavity dilation | Hydronephrosis, renal stones | Nephritic lithiasic colic and hydronephrosis screening |
|  |  | Prostate volume | Evaluation of prostate |  |
|  |  |  | Evaluation of testicles |  |
|  | Varicocele/ Hydrocele | Varicocele |  | Varicocele |
|  |  | Hydrocele |  | Hydrocele |
|  |  | Testicular mass |  |  |
|  |  | Epididymo-orchitis |  |  |

| **Organ system** | **Lokkegaard et al.** | **Camard et al.** | **Homar et al.** | **Conangla-Ferrin et al.** |
| --- | --- | --- | --- | --- |
| **Musculoskeletal** |  |  | Joint effusion | Joint effusion |
|  |  |  | Bursitis | Bursitis |
|  |  |  |  | Tenosynovitis, tendinosis, and calcifications |
|  | Baker’s cyst |  |  |  |
|  | Knee joint effusion |  |  |  |
|  | Achilles tendinitis and tendon rupture |  |  |  |
|  | Trochanter bursitis |  |  |  |
|  | Elbow joint effusion |  |  |  |
|  | Rotator cuff tendinitis and/or ruptures (partial or full) |  |  |  |
|  | Subacromial/ subdeltoid bursitis |  |  |  |
|  | Biceps tendinitis, tenosynovitis, and tendon rupture |  |  |  |
|  |  |  | Injuries: muscle, tendon, rupture, haematoma | Tendon or muscle rupture |
|  |  |  | Fractured bone |  |

| **Organ system** | **Lokkegaard et al.** | **Camard et al.** | **Homar et al.** | **Conangla-Ferrin et al.** |
| --- | --- | --- | --- | --- |
| **Other organs** |  |  | Evaluation of lymph nodes | Adenopathy |
|  | Subcutaneous abscesses |  | Abscess | Abscesses and subcutaneous cysts |
|  | Subcutaneous tumours (lipoma, atheroma) |  |  | Lipoma |
|  |  |  | Unclear subcutaneous tumours | Ultrasound confirmation of solid mass and associated complications |
|  | Localization of foreign body |  | Soft tissue foreign bodies |  |
|  |  | Subcutaneous contraceptive device |  |  |
|  |  |  |  | Thyroid hyperplasia |
|  |  |  |  | Thyroid nodule and ultrasound classification of palpable nodules |
|  |  |  |  | Study of nodule in salivary gland |

| **Organ system** | **Lokkegaard et al.** | **Camard et al.** | **Homar et al.** | **Conangla-Ferrin et al.** |
| --- | --- | --- | --- | --- |
| **Procedural** | Injection shoulder |  |  |  |
|  | Injection/ aspiration knee joint |  |  |  |
|  | Injection/ aspiration, Baker’s cyst |  |  |  |
|  |  |  |  | US guided joint and peri-articular punctures |
|  | Ultrasound guided abscess drainage |  |  | US guided drainage of cysts, abscesses, and bruises |
|  |  |  | Causes of cardiac arrest (4H, 4 T) | Shock/Cardiac arrest – Multiorgan aetiological study including specific protocols: RUSH, SESAME, CAUSE, FATE |
|  |  |  | FAST examination | Polytrauma including free abdominal and thoracic fluid with specific protocols FAST, eFAST |
|  |  |  | Verification of endotracheal tube placement | US guided orotracheal intubation |
|  |  |  |  | US guided cricothyroidotomy |
|  |  |  |  | US guided thoracocentesis and paracentesis |
|  |  |  |  | Acute respiratory failure including specific protocol BLUE |
|  |  |  |  | US guided channelling of venous or arterial routes |
|  |  |  |  | US guided lumbar puncture |
|  |  |  |  | US guided suprapubic probing |

**References**

1. Lokkegaard T, Todsen T, Nayahangan LJ, Andersen CA, Jensen MB, Konge L. Point-of-care ultrasound for general practitioners: a systematic needs assessment. Scand J Prim Health Care. 2020:1-9.

2. Camard L, Liard R, Duverne S, Ibanez G, Skendi M. Consensus on relevant point-of-care ultrasound skills in General Practice: a two-round French Delphi study. BMC Med Educ. 2024;24(1):341.

3. Homar V, Gale ZK, Lainscak M, Svab I. Knowledge and skills required to perform point-of-care ultrasonography in family practice - a modified Delphi study among family physicians in Slovenia. BMC Fam Pract. 2020;21(1):56.

4. Conangla-Ferrin L, Guirado-Vila P, Solanes-Cabús M, Teixidó-Gimeno D, Díez-García L, Pujol-Salud J, et al. Ultrasound in primary care: Consensus recommendations on its applications and training. Results of a 3-round Delphi study. The European journal of general practice. 2022;28(1):253-9.
